# Exploring trauma recovery in nurses: a text mining and thematic analysis based on Swanson’s theory of caring

**DOI:** 10.1186/s12912-025-02757-y

**Published:** 2025-03-12

**Authors:** Jinyoung Park, Goun Kim, Sunah Kim

**Affiliations:** 1 College of Nursing and Mo-Im Kim Nursing Research Institute, Yonsei University, 50-1 Yonsei-ro, Seodaemun-gu, Seoul, 03722 Republic of Korea; 2 College of Nursing and Research Institute of Nursing Science, Pusan National University, 49 Busandaehak-ro, Yangsan-si, 50612 Gyeongsangnam-do Republic of Korea

**Keywords:** Psychological trauma, Psychosocial intervention, Nurses, Swanson’s theory of Caring

## Abstract

**Background:**

In their personal lives and workplace, nurses are exposed to traumatic events, which heighten their risk of developing post-traumatic stress disorder. However, targeted interventions to promote psychological recovery among nurses are limited. This study explored the emotional and psychological recovery processes of nurses who participated in an Internet-based Trauma Recovery Nursing Intervention (IBTRNI) based on Swanson’s Theory of Caring.

**Methods:**

This study conducted a secondary qualitative analysis of free-text responses collected from 102 nurses who completed IBTRNI, as part of a survey-based study with both closed- and open-ended questions. Text mining was utilized to identify high-frequency keywords, and thematic analysis provided deeper emotional and psychological insights. The analysis was structured around Swanson’s three phases: “Knowing,” “Doing For,” and “Enabling.”

**Results:**

In the “Knowing phase,” the participants demonstrated increased self-awareness, recognizing their emotional responses and the effects of negative thoughts on daily life. The “Doing For” phase revealed enhanced emotional regulation, where participants learned to manage and transform negative emotions into positive ones. Finally, the “Enabling” participants developed improved interpersonal relationships and adopted effective coping mechanisms, including communication and mindfulness practices, to manage stress and promote resilience.

**Conclusions:**

Swanson’s Theory of Caring provides a robust framework for supporting nurses’ trauma recovery. The combination of text mining and thematic analysis offers a comprehensive understanding of the emotional and psychological transformations experienced during the intervention. The findings underscore the potential for theory-based digital interventions to support trauma recovery among healthcare professionals. Future research should expand on these methodologies to enhance their broader applicability.

**Trial registration:**

This study involved secondary data analysis. The primary study was registered at ClinicalTrials.gov—US National Library of Medicine (clinical trial registration number: NCT04989582) on 2022-01-31 and is available online.

## Background

Post-traumatic stress disorder (PTSD) is a mental health condition stemming from direct or indirect exposure to traumatic events [1]. Nurses are particularly vulnerable to trauma, not only due to their repeated exposure to critical situations in the workplace, such as caring for patients with physical and psychological trauma, but also due to personal challenges in their private lives [2]. High rates of PTSD have been documented among nurses in critical care fields such as intensive care, emergency care, oncology, and psychiatric nursing [3, 4]. Moreover, in the process of empathetic listening and providing care, nurses face the risk of secondary traumatic stress, further exacerbating their vulnerability [5].

Nurses also experience first-hand trauma driven by the prevalence of workplace violence in healthcare settings. Nurses, due to direct contact with patients and their companions, are 3 times more likely to experience workplace violence compared to other healthcare employees [6, 7]. If left unresolved, the cumulative stress from patient care and workplace abuse could lead to severe mental health issues, including depression, anxiety, and PTSD, significantly impacting job performance, job satisfaction, and personal well-being [8, 9]. Despite these challenges, there are limited interventions aimed at promoting post-traumatic recovery, particularly those tailored for healthcare professionals [10].

Given the significant challenges faced by nurses in managing trauma, implementing effective and accessible interventions, such as online recovery programs, has become imperative [11, 12]. For example, Kim et al. [13] implemented an Internet-based trauma recovery nursing intervention (IBTRNI) based on Swanson’s Theory of Caring [14], which emphasizes understanding individuals’ experiences (Knowing), providing practical support to foster positive emotions (Doing), and helping individuals integrate learned behaviors into daily life (Enabling). This intervention significantly improved nurses’ mental health and resilience through quantitative measures. However, while quantitative findings seem valuable, they often fail to capture the complexity of participants’ experiences. To understand the nuances of such interventions, a combination of quantitative and qualitative approaches is necessary [15].

Traditional qualitative analysis methods such as manual coding, discourse analysis, and grounded theory are time-consuming and labor-intensive, making them inefficient for large-scale data analysis [16]. Automated approaches such as text mining have emerged to address these limitations, efficiently processing large volumes of text data [17]. Abbe et al. [18] emphasized the benefits of integrating text mining with traditional qualitative methods, highlighting its ability to enhance the depth and breadth of insights. However, they also pointed out that text mining alone has limitations, including difficulties capturing the emotional and psychological complexities of participants’ experiences, reliance on large datasets for reliable results, and processing linguistic nuances such as ambiguity and context. To address these limitations, this study combines text mining with thematic analysis to provide a more comprehensive interpretation of the data and uncover latent meanings that automated methods alone may not reveal. Braun and Clarke [19] argue that thematic analysis offers a flexible and systematic approach to qualitative data analysis, making it a valuable method for exploring patterns in psychological research.

Therefore, this study aims to comprehensively analyze nurses’ experiences in IBTRNI using a combination of text mining and thematic analysis, ensuring efficient data processing and comprehensive exploration of participants’ emotional and psychological processes.

## Methods

### Study design

This study employed a secondary qualitative analysis approach to analyze free-text responses from nurses who participated in IBTRNI sessions as part of a survey-based study in a previous study [13]. Text mining, which involves extracting meaningful patterns and insights from large volumes of unstructured text data through computational methods [20], was used to analyze session-based responses. Text mining has been widely applied in healthcare research, such as identifying smoking status from electronic health records or detecting adverse drug events through natural language processing [21, 22]. This study used text mining to extract keywords and generate word cloud visualizations, effectively identifying major patterns within the responses.

Additionally, thematic analysis was conducted to explore the deeper meanings and emotional changes embedded in the responses from each session. The qualitative approach adopted a constructivist paradigm [23] to understand participants’ experiences as shaped by personal meaning-making processes. This allowed for a comprehensive exploration of the emotional and psychological recovery process, recognizing subjective experiences as socially constructed.

### Participants

Participants in this study included 102 general nurses. Initially, 112 nurses were recruited for the primary study, but the secondary analysis included only 102 nurses who completed the intervention. Participants were sourced from two prominent online nursing platforms in Korea. The first, “Meetings Representing Nurses,” is the country’s largest nursing community, boasting over 95,000 registered members and averaging approximately 12,700 daily active users. The second platform, “I am a Nurse,” includes around 35,000 registered members, with an average daily activity of 150 users.

The inclusion criteria (applied in the primary study) were: (1) aged 23–40, (2) self-score greater than 80% (64 points) on the Korean version of the PTSD Checklist for DSM-5 (PCL-5), which evaluates overall trauma symptoms across four symptom clusters, (3) ability to access the program through a mobile device, (4) understand the purpose of the study and voluntarily agree to participate, and (5) Participants were excluded if they had been diagnosed with a severe mental illness and were taking medication for its treatment, even if they met the PTSD score threshold (≥ 64 points).

The demographic characteristics of the participants are summarized in Table 1. The mean age of participants was 31.04 (SD = 4.50), ranging from 22 to 40. Females accounted for most participants (96.1%), and the distribution of religious affiliation was evenly split, with 50% identifying themselves as religious. Most participants were single (60.8%), with 38.2% married and 1.0% separated, widowed, or divorced. Regarding educational background, 75.5% had attained a college degree or higher, and 24.5% had completed graduate school. The vast majority (90.2%) were currently employed, with 33.3% having 5–10 years of work experience and 27.5% having ten or more years of experience. Additionally, regarding the type of organization, 45 (44.1%) worked in tertiary referral hospitals, 52 (50.0%) in hospitals, and 5 (4.9%) in other organizations, including schools and health centers.

**Table 1:** Demographic characteristics of the participants (*N* = 102)

Variables	Categories	*N* (%)	Mean (SD)
Age (years)			31.04 (4.50) (Range: 22–40)
Gender			
	Men	4 (3.9)	
	Women	98 (96.1)	
Religion			
	Yes	51 (50.0)	
	No	51 (50.0)	
Marital status			
	Single	62 (60.8)	
	Married	39 (38.2)	
	Separated, widowed, or divorced	1 (1.0)	
Education level			
	College or higher	77 (75.5)	
	Graduate school or higher	25 (24.5)	
Current employment			
	Current	92 (90.2)	
	None or past	10 (9.8)	
Working experience			
	< 3 years	19 (18.6)	
	3–< 5 years	21 (20.6)	
	5–< 10 years	34 (33.3)	
	≥ 10 years	28 (27.5)	

### Intervention: internet-based trauma recovery nursing intervention (IBTRNI)

The IBTRNI was developed by the primary research team using Swanson’s theoretical framework [14, 24]. The secondary analysis analyzed these sessions through session-specific questions, focusing on three key concepts: “Knowing,” “Doing For,” and “Enabling.” ***Knowing*** emphasized self-understanding and self-respect, ***Doing For*** focused on self-acceptance and releasing negative emotions, and ***Enabling*** centered on effective communication and stress management [13, 24].

Nurses participating in the study responded to these questions at the end of each session in the online program. Responses were provided through text entries, allowing participants to explore their thoughts and feelings in relation to the goals of individual sessions. The data were automatically collected and stored by the platform. Researchers communicated with the participants throughout the intervention by providing feedback after each session and offering ongoing emotional support. Researchers provided participants standardized response letters after each session to ensure consistent feedback and emotional support throughout the intervention. The research team carefully developed these letters to align with the program’s goals and minimize the influence of individual researcher biases. While researchers maintained open communication to support participants, all interactions adhered to predefined protocols to reduce the potential for subjective influence. At the end of each section, informed by Swanson’s Theory of Caring, the participants were allowed to express their experiences and reflections on how they had changed through structured reflective questions. These reflective questions were designed to encourage nurses to assess their emotional and psychological changes during recovery, allowing for personal and subjective insights. For a detailed overview of these theory-based questions, refer to Table 2.

**Table 2:** Theory-based questions in the IBTRNI Program

Theory (Session)	Question
Knowing (1 ~ 2)	What new things did you learn about yourself, and what do you expect from yourself?
	How is that emotional distress affecting your current daily life?
Doing (3 ~ 5)	What did you learn or what was helpful to you during this session?
	What can you do to experience more positive emotions in your daily life?
Enabling (6 ~ 8)	Over the past week, was there a situation where you believe you handled stress well? If so, describe the situation and how you managed it.
	After completing this program, what would you like to continue applying it to your daily life?

### Data collection and ethical considerations

A secondary analysis was conducted using data from a primary study [13]. Institutional Review Board (IRB) approval (IRB No. Y-2021-0790) from X-University (blinded for review) was obtained for the secondary analysis. Participants in the primary study were informed of the current study and provided consent for using their responses in secondary data analysis.

### Data analysis

We employed text mining and thematic analysis to systematically analyze nurses’ responses. For text mining, we used R (version 4.2.2), a widely-recognized tool for processing unstructured data [25]. The KoNLP (Korean Natural Language Processing) package and NIADic dictionary are well-established tools for processing Korean text [17]; however, they may have limitations in capturing certain contextual nuances specific to this study’s dataset [18]. To ensure accurate analysis, the research team conducted a heuristic purification process. This method focuses on iterative refinement to enhance the reliability of key issue extraction. By repeating the heuristic purification process, analysts can improve the reliability of the extracted key issues and address contextual nuances and inconsistencies to derive meaningful insights [26]. Contextually significant terms that were not included in the dictionary (e.g., “I-message”) were incorporated based on the consensus of the research team, ensuring their relevance to the study objectives. Common data pre-processing steps were taken, including eliminating duplicate and meaningless words (e.g., copulas, adjectives, adverbs) by designating them as stop words. Words that did not contribute to the overall context or analysis, such as frequently occurring function words (e.g., “있다,” “하다”), were also excluded. In cases where contextually-significant terms did not appear in the dictionary (e.g., “I-message”), the research team reached a consensus to include them as unique terms based on their relevance to trauma recovery and emotional processing themes. The cleaned data was then visualized using ggplot, through bar graphs and word clouds, to identify key patterns and high-frequency keywords.

To complement the text mining analysis, thematic analysis followed the six-phase approach outlined by Braun and Clarke [19]. Text mining identified high-frequency words from the participants’ responses, and corresponding sentences were subsequently extracted for further qualitative analysis. The researchers familiarized themselves with the extracted sentences through repeated reviews. Three team members independently performed initial coding, systematically identifying meaningful text segments. The coding process facilitated using Excel, breaking down each response into meaningful units and assigning descriptive codes to capture recurring patterns. Subsequently, the researchers convened to resolve any discrepancies in coding, ensuring inter-coder reliability. Once a consensus was reached, the codes were grouped into overarching themes that accurately reflected nurses’ emotional and psychological experiences during the intervention. These broader themes were reviewed iteratively to ensure consistency and alignment with the study objectives. The thematic analysis was conducted within the framework of Swanson’s Caring Theory, which served as the basis for both intervention design and analysis of participants’ responses. This theoretical approach guided the coding and categorization of themes throughout the analysis. Swanson’s three key concepts—“Knowing,” “Doing For,” and “Enabling”—were employed to categorize participants’ responses, facilitating the evaluation of how well the intervention aligned with the intended theoretical goals.

## Results

### Keyword and thematic analyses of trauma recovery experiences: applying Swanson’s theory of Caring

**Fig. 1:**
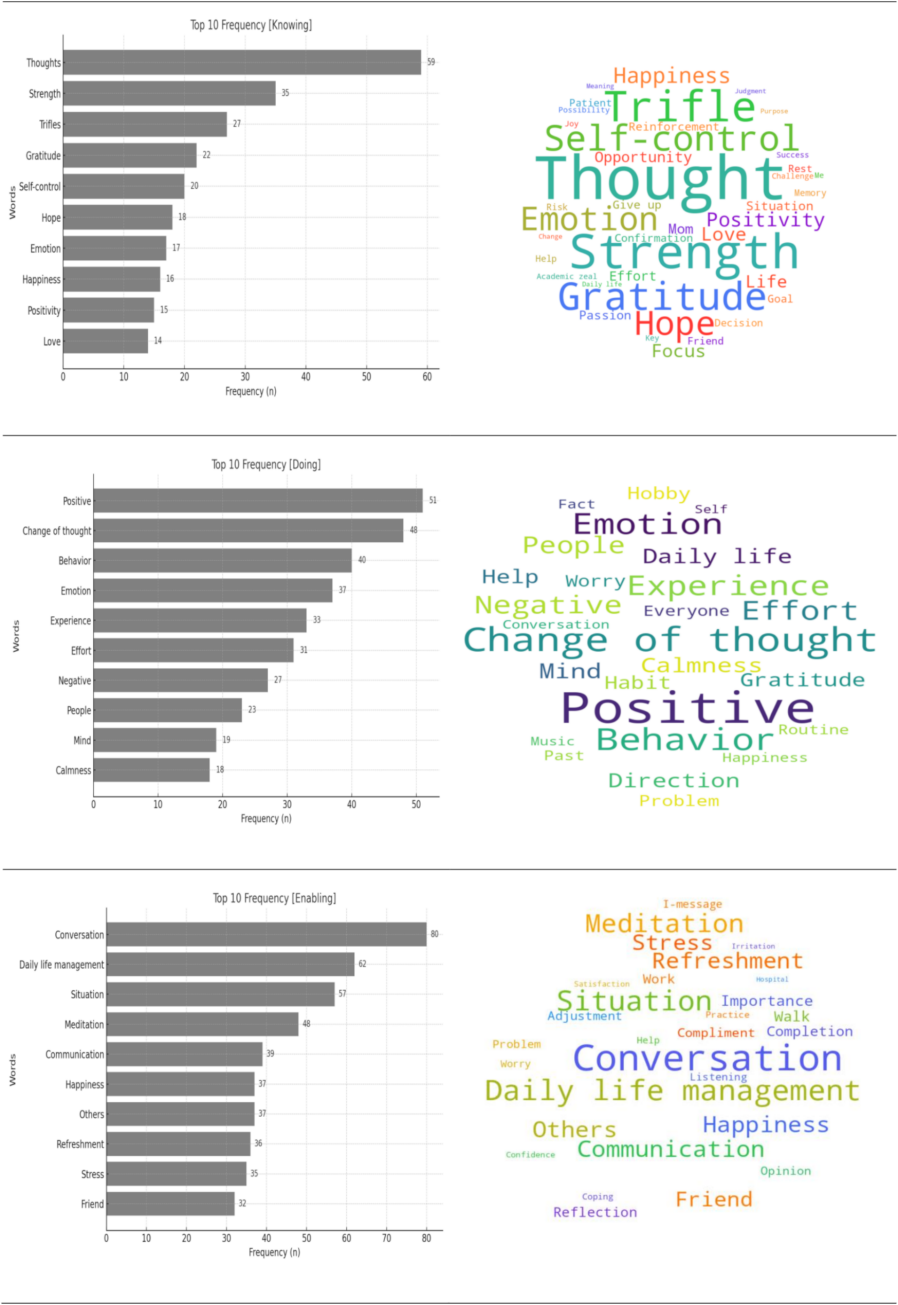
Visualization of keyword analysis: top 10 keywords and word cloud

This study explored the trauma recovery experiences of nurses who participated in the IBTRINI program. Participants’ free-text responses were analyzed using a combination of text mining and thematic analysis based on Swanson’s three key concepts: “Knowing,” “Doing For,” and “Enabling.” Fig. 1 illustrates the top 10 keywords identified at each stage of the program, representing the most frequently mentioned terms by the participants during each phase. In addition to bar charts, word clouds visually depict the relative frequency of these keywords.

### The knowing phase (Session 1–2)

The “Knowing” phase focused on enhancing self-awareness and recognizing personal strengths. Text mining identified keywords such as thoughts, strength, trifles, gratitude, and self-control, reflecting participants’ focus on their emotional state and personal development.

### Theme: self-awareness

The participants reported an increased awareness of their emotional responses and thought patterns, which facilitated the recognition of the impact of negative thoughts on their daily lives.

*“I didn’t realize how many negative thoughts I had until this session made me aware of them.”*

*“I always believed that I was strong*,* but through this program*,* I realized how emotionally vulnerable I was.”*

Additionally, the participants expressed a newfound appreciation of their strengths and the smaller, often overlooked, positive aspects of their lives, which contributed to their emotional resilience.

*“I now try to appreciate even the small things in my life*,* and that’s a huge change for me.”*

*“Reflecting on my strengths made me appreciate even the trifles that I used to overlook.”*

### The doing for phase (Session 3–5)

The “Doing For” phase centered on emotional regulation and behavioral transformation. Text mining revealed keywords such as positive, change of thought, behavior, emotion, and experience, indicating participants’ efforts to manage emotional responses and modify their behaviors.

### Theme: emotional regulation

The participants learned strategies to manage negative emotions and shift their responses toward more positive outcomes. Emotional regulation was crucial for behavioral changes and stress management.

*“I started focusing more on positive thoughts and how they influenced my emotions*,* which helped me manage stress.”*

*“One of the biggest changes was learning to shift my emotions from negative to positive and recognizing how that impacted my behavior.”*

By practicing emotional regulation, participants gained better control over their behaviors, allowing them to handle stress effectively and respond proactively in challenging situations.

*“Now*,* when I face stressful situations*,* I change my thoughts and behavior before reacting emotionally.”*

*“This experience taught me how to control my emotions and modify my behavior in difficult situations.”*

### The enabling phase (Session 6–8)

In the “Enabling” phase, the participants developed improved communication skills and stress management techniques. Text mining identified keywords such as conversation, daily life management, situation, meditation, and communication, focusing on managing interpersonal relationships and daily stressors.

### Theme: interpersonal relationships

The participants reported significant improvements in their interpersonal relationships, facilitated by enhanced communication skills and effective stress management strategies. Meditation was frequently mentioned as a tool that helped participants regulate emotions and engage more positively with others.

*“Through meditation*,* I’ve been able to control my emotions*,* and it’s made conversations with others much easier.”*

*“I’ve learned not to always be defensive in conversations*,* which has improved my relationships.”*

Moreover, the participants reported better management of daily life and stress, which empowered them to easily navigate challenging situations.

*“Stress used to overwhelm me*,* but now I manage it through meditation and self-care.”*

*“I’ve started managing my daily life effectively*,* and when stressful situations arise*,* I feel more in control.”*

## Discussion

This study employed text mining and thematic analysis to explore the emotional and psychological recovery processes of nurses who participated in an IBTRNI guided by Swanson’s Theory of Caring. Text mining efficiently processed large volumes of data to identify key patterns, while thematic analysis facilitated a nuanced understanding of participants’ experiences, particularly their emotional regulation and stress management strategies.

In the “Knowing” phase, the dominant theme of self-awareness emerged. Text mining revealed keywords such as thoughts, strength, gratitude, and self-control, reflecting participants’ increased awareness of their emotional state and personal development. This self-awareness is critical in trauma recovery, as recognizing the impact of negative thoughts on daily life is a vital step toward emotional resilience [27, 28]. Prior research supports the importance of self-reflection in promoting emotional recovery, where acknowledging personal strengths and practicing gratitude have been shown to alleviate symptoms of depression and anxiety, which are often associated with trauma, thereby supporting the process of emotional recovery [29]. Swanson’s concept of “Knowing” emphasizes the value of self-understanding and self-respect, which aligns closely with these findings [14].

The “Doing For” phase focused on emotional regulation and behavioral transformation, central themes identified through keywords and thematic analysis. Keywords such as positive thinking, behavioral change, and emotional control were highlighted, illustrating the cognitive and emotional shifts that participants experienced. This aligns with previous studies that emphasized the role of cognitive restructuring in trauma recovery [30–32]. Cognitive restructuring has been shown to significantly reduce PTSD symptoms and enhance functional recovery in individuals experiencing trauma-related difficulties [30, 31]. Through this phase, participants successfully managed their emotions, transitioning from negative to positive emotional responses, which facilitated meaningful behavioral changes.

Cognitive restructuring is also recognized as a critical self-management skill that enables individuals to articulate distressing thoughts, evaluate their validity, and implement adaptive coping mechanisms [32]. It has been particularly effective in promoting emotional stability and resilience in trauma-exposed populations, addressing both emotional under- and overmodulation to facilitate long-term recovery. These processes complement the goals of the “Doing For” phase, which actively supports participants in managing their emotions and developing effective behavioral strategies. This approach is consistent with Swanson’s theory, where “Doing For” involves nurturing actions that actively enhance individuals’ emotional and psychological well-being.

The “Enabling” phase in this study revealed interpersonal relationships and stress management themes. Keywords such as conversation, meditation, and stress management were identified, reflecting participants’ application of learned coping mechanisms to manage stress and improve social connections. This aligns with existing research highlighting the importance of communication and stress management strategies in promoting emotional stability during trauma recovery [33, 34]. Interpersonal relationships, a critical component of recovery, rely on effective communication and social support. Studies indicate that individuals with PTSD often experience difficulties in social cognition, which can hinder meaningful interactions and emotional connection [35, 36]. This study’s findings suggest that participants improved their ability to navigate interpersonal challenges and build supportive relationships, consistent with the goals of the “Enabling” phase in Swanson’s theory. Furthermore, participants’ use of stress management techniques, including meditation, aligns with prior evidence demonstrating the efficacy of such approaches in reducing PTSD symptoms and enhancing emotional regulation [37, 38]. These strategies likely contributed to improved coping and overall well-being during the intervention. This phase highlights the potential of structured, theory-based interventions to address both social and emotional aspects of recovery, supporting participants in managing stress and fostering resilience through practical and relational strategies.

### Strengths

The combination of text mining and thematic analysis can be considered a robust methodological approach for examining the complex nature of trauma recovery [18]. Text mining efficiently identified key patterns across large datasets, while thematic analysis provided richer insights into the emotional and psychological transformations experienced by participants. The integration of these methods approaches validates the relevance of Swanson’s theory in nursing interventions and offers a scalable framework for investigating trauma recovery processes across diverse healthcare settings.

### Implications for nursing practice

The findings of this study underscore the practical applicability of IBTRNI in supporting nurses’ psychological recovery from trauma. By fostering the development of stress management techniques and communication skills, the intervention enhanced emotional resilience and coping abilities, which are critical for restoring psychological balance and functionality disrupted by trauma. These improvements highlight the potential of such interventions to promote emotional stability and overall well-being during recovery.

Additionally, this study emphasizes the necessity of integrating psychological recovery and coping strategy training into nursing education curricula. Preparing nurses with the skills to effectively manage the psychological impacts of trauma will contribute to building a more resilient healthcare workforce. Future research should focus on adapting IBTRNI for implementation in diverse clinical and cultural contexts, maximizing its impact on trauma recovery and psychological well-being.

### Limitations

This study has several limitations. First, the relatively small sample size (*n* = 102) limits the generalizability of the findings to a broader population. Also, the study focused on nurses aged 23–40 years to explore the experiences of early to mid-career nurses in trauma recovery. However, excluding later-career nurses may limit the generalizability of the findings, which is acknowledged as a limitation of this study. Second, the KoNLP and NIADic dictionaries may not fully capture the subtle contextual nuances of the Korean language, necessitating additional manual validation. However, the manual process may introduce potential bias in data analysis. Therefore, to mitigate such potential bias, this study also employed thematic analysis to identify context-specific meanings and derive key themes. Additionally, since this study focused exclusively on Korean nurses, there may be limitations in applying the results directly to other populations or cultural contexts. Similarly, this study did not collect specific information regarding the clinical settings or professional specialties of participants, which may limit a nuanced understanding of how these factors influence trauma experiences and recovery. Future research should aim to include more diverse populations and examine the role of various clinical settings or professional specialties to enhance the generalizability and applicability of the findings.

## Conclusions

This study demonstrates that the trauma recovery program, based on Swanson’s Theory of Caring, effectively aligned with the recovery experiences of the participants. Text mining revealed key patterns, while thematic analysis provided deeper insights into the emotional transformations of nurses. The observed changes in the participants closely corresponded with Swanson’s theoretical framework, particularly within the “Knowing,” “Doing For,” and Enabling phases. These findings suggest that the theoretical model effectively guided and supported the mental health recovery of the participants. Future research should focus on refining these methodologies and expanding the sample to validate the broader applicability of the intervention.

## Data Availability

The datasets generated and/or analyzed during the current study are available from the corresponding author upon reasonable request.

## Abbreviations

PTSD: Post-traumatic stress disorder
IBTRNI: Internet-based trauma recovery nursing intervention
KoNLP: Korean Natural Language Processing
